# In Vivo Reactive Astrocyte Imaging in Patients With Schizophrenia Using Fluorine 18–Labeled THK5351

**DOI:** 10.1001/jamanetworkopen.2024.10684

**Published:** 2024-05-09

**Authors:** Minah Kim, Woori Choi, Sunah Choi, Harin Oh, Jongrak Kim, Jungha Lee, Su-Jin An, Jun Seo Hwang, Yun-Sang Lee, In Chan Song, Sun-Young Moon, Silvia Kyungjin Lho, Sang Soo Cho, Jun Soo Kwon

**Affiliations:** 1 Department of Neuropsychiatry, Seoul National University Hospital, Seoul, Republic of Korea; 2 Department of Psychiatry, Seoul National University College of Medicine, Seoul, Republic of Korea; 3 Department of Brain and Cognitive Sciences, Seoul National University College of Natural Sciences, Seoul, Republic of Korea; 4 Department of Nuclear Medicine, Seoul National University College of Medicine, Seoul, Republic of Korea; 5 Department of Radiology, Seoul National University College of Medicine, Seoul, Republic of Korea; 6 Department of Public Health Medical Services, Seoul National University Bundang Hospital, Seongnam, Republic of Korea; 7 Department of Psychiatry, Seoul Metropolitan Government–Seoul National University Boramae Medical Center, Seoul, Republic of Korea; 8 Institute of Human Behavioral Medicine, Seoul National University–Medical Research Center, Seoul, Republic of Korea

## Abstract

**Importance:**

In vivo imaging studies of reactive astrocytes are crucial for understanding the pathophysiology of schizophrenia because astrocytes play a critical role in glutamate imbalance and neuroinflammation.

**Objective:**

To investigate in vivo reactive astrocytes in patients with schizophrenia associated with positive symptoms using monoamine oxidase B (MAO-B)–binding fluorine 18 ([^18^F])–labeled THK5351 positron emission tomography (PET).

**Design, Setting, and Participants:**

In this case-control study, data were collected from October 1, 2021, to January 31, 2023, from the internet advertisement for the healthy control group and from the outpatient clinics of Seoul National University Hospital in Seoul, South Korea, for the schizophrenia group. Participants included patients with schizophrenia and age- and sex-matched healthy control individuals.

**Main Outcomes and Measures:**

Standardized uptake value ratios (SUVrs) of [^18^F]THK5351 in the anterior cingulate cortex (ACC) and hippocampus as primary regions of interest (ROIs), with other limbic regions as secondary ROIs, and the correlation between altered SUVrs and Positive and Negative Syndrome Scale (PANSS) positive symptom scores.

**Results:**

A total of 68 participants (mean [SD] age, 32.0 [7.0] years; 41 men [60.3%]) included 33 patients with schizophrenia (mean [SD] age, 32.3 [6.3] years; 22 men [66.7%]) and 35 healthy controls (mean [SD] age, 31.8 [7.6] years; 19 men [54.3%]) who underwent [^18^F]THK5351 PET scanning. Patients with schizophrenia showed significantly higher SUVrs in the bilateral ACC (left, *F* = 5.767 [false discovery rate (FDR)–corrected *P* = .04]; right, *F* = 5.977 [FDR-corrected *P* = .04]) and left hippocampus (*F* = 4.834 [FDR-corrected *P* = .04]) than healthy controls. Trend-level group differences between the groups in the SUVrs were found in the secondary ROIs (eg, right parahippocampal gyrus, *F* = 3.387 [*P* = .07]). There were positive correlations between the SUVrs in the bilateral ACC and the PANSS positive symptom scores (left, *r* = 0.423 [FDR-corrected *P* = .03]; right, *r* = 0.406 [FDR-corrected *P* = .03]) in patients with schizophrenia.

**Conclusions and Relevance:**

This case-control study provides novel in vivo imaging evidence of reactive astrocyte involvement in the pathophysiology of schizophrenia. Reactive astrocytes in the ACC may be a future target for the treatment of symptoms of schizophrenia, especially positive symptoms.

## Key Points

**Question:**

Can region-specific reactive astrocytes in vivo associated with positive symptoms in patients with schizophrenia be measured using fluorine 18–labeled THK5351 ([^18^F]THK5351) positron emission tomography?

**Findings:**

In this case-control study of 68 participants, standardized uptake value ratios (SUVrs) of [^18^F]THK5351 in the bilateral anterior cingulate cortex and left hippocampus were greater in patients with schizophrenia than in healthy controls. Increased [^18^F]THK5351 SUVrs were correlated with positive symptom severity in patients with schizophrenia.

**Meaning:**

Considering the role of astrocytes in brain development, neurotransmission, and immune reactions, reactive astrocytes in the bilateral anterior cingulate cortex and left hippocampus may be strong biomarkers for schizophrenia pathophysiology and treatment.

## Teaser

This case-control study investigates the in vivo imaging of reactive astrocytes and their association with positive symptoms in patients with schizophrenia using validated monoamine oxidase B–binding fluorine 18–labeled THK5351 ([^18^F]THK5351) positron emission tomography.

## Introduction

Glutamatergic imbalance and neuroinflammation are believed to be important in schizophrenia pathophysiology. The glutamate hypothesis suggests that psychotic symptoms are caused by *N*-methyl-d-aspartate receptor hypofunction-mediated abnormal glutamatergic neurotransmission with a dysfunctional thalamic filter system.^1,2,3^ Furthermore, excessive glutamate activity might lead to excitotoxic damage and oxidative stress–related neuroinflammation, which further explains the pathophysiology.^4,5^ This finding is consistent with the immune hypothesis suggesting the involvement of neuroinflammation caused by microglial overactivation in the development and progression of schizophrenia.^6,7,8,9^

Previous magnetic resonance (MR) spectroscopy studies have revealed that patients with schizophrenia exhibit altered glutamate and/or glutamine levels in several brain regions, including the anterior cingulate cortex (ACC) and hippocampus, although the direction and degree of alterations have been inconsistent.^10,11,12,13^ Among individuals at clinically high risk for psychosis, the baseline hippocampal glutamate level was suggested to be associated with the transition to psychotic disorder.^14^ In vivo microglial imaging studies using 18-kDa translocator protein positron emission tomography (PET) to detect neuroinflammation in the frontal cortex, ACC, temporal cortex, and hippocampus of patients with schizophrenia have also provided inconsistent results.^15,16,17,18^ These inconsistencies may be due to the simple approach of using individual glutamate or neuroinflammation markers in a rather complex, mutually influencing system. However, investigations aimed at integrating glutamate imbalance and neuroinflammation in the pathophysiology of schizophrenia are limited, and only 1 study^5^ has attempted to bridge the gap between the 2 hypotheses by showing that the levels of the antioxidant glutathione and excitotoxic glutamate and/or glutamine are lower in patients with schizophrenia who are in stable clinical condition.

Reactive astrocytes are promising candidates for achieving a comprehensive understanding of glutamate imbalance and neuroinflammation in the pathophysiology of schizophrenia because astrocytes play an important role in glutamate recycling, neurotransmission (including dopamine), and the neuroimmune system, in addition to their basic role in supporting neurons.^19,20,21^ Reactive astrocytes are remodeled in response to injury, disease, or infection of the brain and can be measured in vivo by detecting overexpressed monoamine oxidase B (MAO-B) on the outer mitochondrial membrane.^22^ In patients with schizophrenia, abnormal astrocyte-neuronal interactions have been suggested to be the mechanism of psychotic symptom development,^2^ and alterations in the expression of astrocyte-related genes and their products in patients’ postmortem brains have been reported.^23^ However, in vivo reactive astrocyte imaging has not yet been reported in patients with schizophrenia.

In this study, we investigated the in vivo imaging of reactive astrocytes and their association with positive symptoms in patients with schizophrenia using validated MAO-B–binding fluorine 18 ([^18^F])–labeled THK5351 PET^24,25,26^ to obtain a more comprehensive understanding of the role of reactive astrocytes in schizophrenia pathophysiology. The primary regions of interest (ROIs) were the ACC and hippocampus based on previous studies of glutamate imbalance, neuroinflammation, and positive symptom development in patients with schizophrenia.^10,13,16,27^ The secondary ROIs included other limbic regions, such as the posterior cingulate cortex (PCC), parahippocampal gyrus, amygdala, insula, and nucleus accumbens, based on previous studies^28,29^ that reported the association between glutamate alterations and positive symptoms in these regions in patients with schizophrenia.

## Methods

This case-control study followed the Strengthening the Reporting of Observational Studies in Epidemiology (STROBE) reporting guideline. All participants provided written informed consent after receiving a thorough explanation of the study procedure. The study was conducted in accordance with the Declaration of Helsinki^30^ and was approved by the Institutional Review Board of Seoul National University Hospital.

### Participants

 A total of 33 patients with schizophrenia and 35 age- and sex-matched healthy controls participated in this study. All study participants were of East Asian descent. Information regarding the sample size calculation is provided in the eMethods in Supplement 1. Patients with schizophrenia were recruited from the outpatient office of the Department of Neuropsychiatry at Seoul National University Hospital. The diagnosis of schizophrenia was confirmed using the Structured Clinical Interview for the *Diagnostic and Statistical Manual of Mental Disorders* (Fourth Edition) Axis I Disorders (SCID-I) by board-certified psychiatrists (M.K., S.-Y.M., and S.K.L.). Psychotic symptoms were assessed using the Positive and Negative Syndrome Scale (PANSS).^31^ The Hamilton Rating Scale for Depression^32^ and the Hamilton Rating Scale for Anxiety^33^ were used to measure the severity of depression and anxiety, respectively. The healthy controls were recruited via internet advertisement and were screened using the SCID-I Nonpatient Edition. Healthy controls were excluded if they had any past or current diagnosis of a psychiatric disorder and any first- to third-degree biological relatives with a psychotic disorder. In all participants, general functional status was evaluated using the modified Global Assessment of Functioning, and intelligent quotient (IQ) was measured with the Korean version of the Wechsler Adult Intelligence Scale.^34^ The exclusion criteria included substance abuse or dependence (except nicotine), neurological disease or significant head trauma, medical illness that could accompany psychiatric symptoms, and intellectual disability (IQ < 70).

### PET-MR Image Acquisition

A PET-MR machine (Biograph mMR; Siemens Healthcare) was used to obtain dynamic 3-dimensional PET images. Immediately after an intravenous bolus injection of 185 MBq (5 mCi) of [^18^F]THK5351, 27 frames of emission scans (8 × 15 seconds, 3 × 60 seconds, 5 × 120 seconds, and 11 × 300 seconds) and a total 70-minute PET scan were acquired while the participant was at rest. Fluorine 18–labeled THK5351 was synthesized and radiolabeled at Seoul National University Hospital, and details are provided in the eMethods in Supplement 1. Each participant was fitted with an MR imaging coil and supporting cushion to reduce head motion during the PET scan, and the participants were asked to remain as still as possible during the scan.

Manufacturer’s software from the PET-MR device (e7tool; Siemens Healthcare) was used for the reconstruction of the PET data. The PET images were reconstructed using the ordered-subset expectation maximization algorithm with 24 subsets and 5 iterations. Images were filtered with a 4-mm full-width at half-maximum Gaussian filter at the center of the field of view (image matrix, 256 × 256; 127 sections; voxel size, 1.4 × 1.4 × 2.0 mm). Segmentation-based attenuation correction was conducted with a 3-tissue segmentation map acquired by an ultrashort echo time (TE) sequence (repetition time [TR], 11.9 milliseconds; TE 1, 0.07 milliseconds; TE 2, 2.46 milliseconds; flip angle, 10°; 192 × 192 matrix). A high-resolution structural T1 image (TE, 2.2 milliseconds; TR, 2400 milliseconds; flip angle, 8°; 0.85-mm section thickness) was also collected for each participant at the same time to rule out structural lesions in the brain and to provide an anatomical reference for the [^18^F]THK5351 analysis.

### PET Image Analysis

All preprocessing was conducted using Statistical Parametric Mapping (SPM 12; Welcome Department of Imaging Neuroscience). The analysis flowchart and selected cerebellar lobules are presented in eFigure 1 in Supplement 1.

For semiquantitative PET analysis, standardized uptake value ratios (SUVrs) were calculated in reference to the inferior cerebellar parcels of the cerebellar cortex. To delineate the inferior cerebellar ROI, the SUIT (spatially unbiased infratentorial template) toolbox,^35^ which contains a high-resolution atlas template of the cerebellum and brainstem and individual T1 images, was used. The cerebellar structure was isolated from the cerebral structure and segmented into tissue types using the Dartel algorithm. Using the deformation field obtained during the Dartel procedure, the SUIT template was transferred to an individual PET space. The lobular ROIs that corresponded to the inferior cerebellar gray matter (bilateral Crus II, VIIb, VIIIa, VIIIb, and IX) were used to extract radioactivity from the PET image. After frame-by-frame motion correction of the PET image, the T1 images were coregistered to the mean images of 27 realigned PET frames.

The bilateral ACC and hippocampus, as primary ROIs, and other limbic regions (bilateral PCC, parahippocampal gyri, amygdala, insula, and nucleus accumbens), as secondary ROIs, were predefined using the Wake Forest University PickAtlas toolbox in SPM 12.^36^ The predefined ROIs were transformed into PET standard space using the deformation matrix calculated from PET-coregistered T1 images, and SUVs were extracted for all PET frames to assess the time activity curve in each ROI. Finally, based on previous studies that tested the optimal time windows to estimate [^18^F]THK5351 quantification,^37^ the SUVr was calculated as the sum of 45- to 65-minute postinjection frames using the mean radioactivity of the inferior cerebellar ROI obtained by the SUIT procedure as a reference.

### Statistical Analysis

Data were collected from October 1, 2021, to January 31, 2023. Demographic and clinical characteristics were compared between patients with schizophrenia and healthy controls using an independent *t* test or a Welch *t* test if the variance was not equal and a χ^2^ test or a Fisher exact test for categorical data. Group differences in the SUVr in the bilateral ACC and hippocampus (ie, primary ROIs) were tested using analysis of covariance, with age and sex as covariates. Pearson correlation analysis was performed to investigate the association between altered SUVrs in primary ROIs and PANSS positive symptom scores in patients with schizophrenia. To rule out the possible effect of the duration of illness or olanzapine-equivalent dose of antipsychotics prescribed at the time of study participation on the SUVrs of the primary ROIs, Pearson correlation analysis was performed. To account for multiple comparisons, a false discovery rate (FDR) correction was performed. Group differences in SUVrs in other limbic ROIs were assessed using repeated-measures analysis of variance (ANOVA), with brain regions (ie, bilateral PCC, parahippocampal gyri, amygdala, insula, and nucleus accumbens) as the within-participants factor and age and sex as covariates. All statistical analyses were performed in SPSS, version 25.0 for Windows (IBM Corporation), and the level of statistical significance was set at 2-sided *P* < .05.

## Results

A total of 68 participants (mean [SD] age, 32.0 [7.0] years; 41 men [60.3%] and 27 women 39.7%]) included 33 patients with schizophrenia (mean [SD] age, 32.3 [6.3] years; 22 men [66.7%] and 11 women [33.3%]) and 35 healthy controls (mean [SD] age, 31.8 [7.6] years; 19 men [54.3%] and 16 women [45.7%]). Table 1 summarizes the demographic and clinical characteristics of the participants. There were no significant group differences in age or sex, while mean (SD) IQ (107.2 [12.5] vs 116.7 [11.0]; *P* = .001) and modified Global Assessment of Functioning scores (54.1 [11.9] vs 87.3 [4.8]; *P* < .001) were lower in patients with schizophrenia than in healthy controls. There were no significant group differences in the amount of [^18^F]THK5351 injected.

**Table 1.** Demographic and Clinical Characteristics of the Participants

Characteristic	Participant group^a^		Statistical analysis	
	Patients with schizophrenia (n = 33)	Healthy controls (n = 35)	χ^2^ or *t* test^b^	*P* value
Sex, No. men/women	22/11	19/16	1.088	.30
Handedness, No. right/left	29/4	35/0	2.584	.11
Age, y	32.3 (6.3)	31.8 (7.6)	0.296	.77
Intelligence quotient	107.2 (12.5)	116.7 (11.0)	−3.362	.001^c^
Duration of illness, mo	160.3 (74.0)	NA	NA	NA
PANSS scores^d^				
Total	49.2 (12.0)	NA	NA	NA
Positive symptoms	12.7 (5.3)	NA	NA	NA
Negative symptoms	11.5 (4.3)	NA	NA	NA
General symptoms	25.0 (5.7)	NA	NA	NA
HAM-A score^e^	3.6 (2.7)	NA	NA	NA
HAM-D score^f^	5.2 (3.4)	NA	NA	NA
mGAF score^g^	54.1 (11.9)	87.3 (4.8)	−15.204	<.001^c^
Antipsychotics^h^	21.5 (14.1)	NA	NA	NA
Injected dose of [^18^F]THK5351, mCi	5.8 (0.6)	5.7 (0.4)	0.656	.51

Abbreviations: HAM-A, Hamilton Rating Scale for Anxiety; HAM-D, Hamilton Rating Scale for Depression; mGAF, modified Global Assessment of Functioning; NA, not applicable; PANSS, Positive and Negative Syndrome Scale.

^a^ Unless otherwise indicated, data are expressed as mean (SD).

^b^ Calculated using an independent *t* test or a Welch *t* test if the variances were not equal, and a χ^2^ test or a Fisher exact test for categorical data.

^c^ Statistically significant at *P* < .005.

^d^ Scores range from 30 to 210, with higher scores indicating greater severity of psychotic symptoms.

^e^ Scores range from 0 to 56, with higher scores indicating greater severity of anxiety.

^f^ Scores range from 0 to 52, with higher scores indicating greater severity of depression.

^g^ Scores range from 1 to 100, with higher scores indicating higher functioning.

^h^ Mean olanzapine equivalent dose of antipsychotics prescribed at the time of study participation.

**Figure 1.**
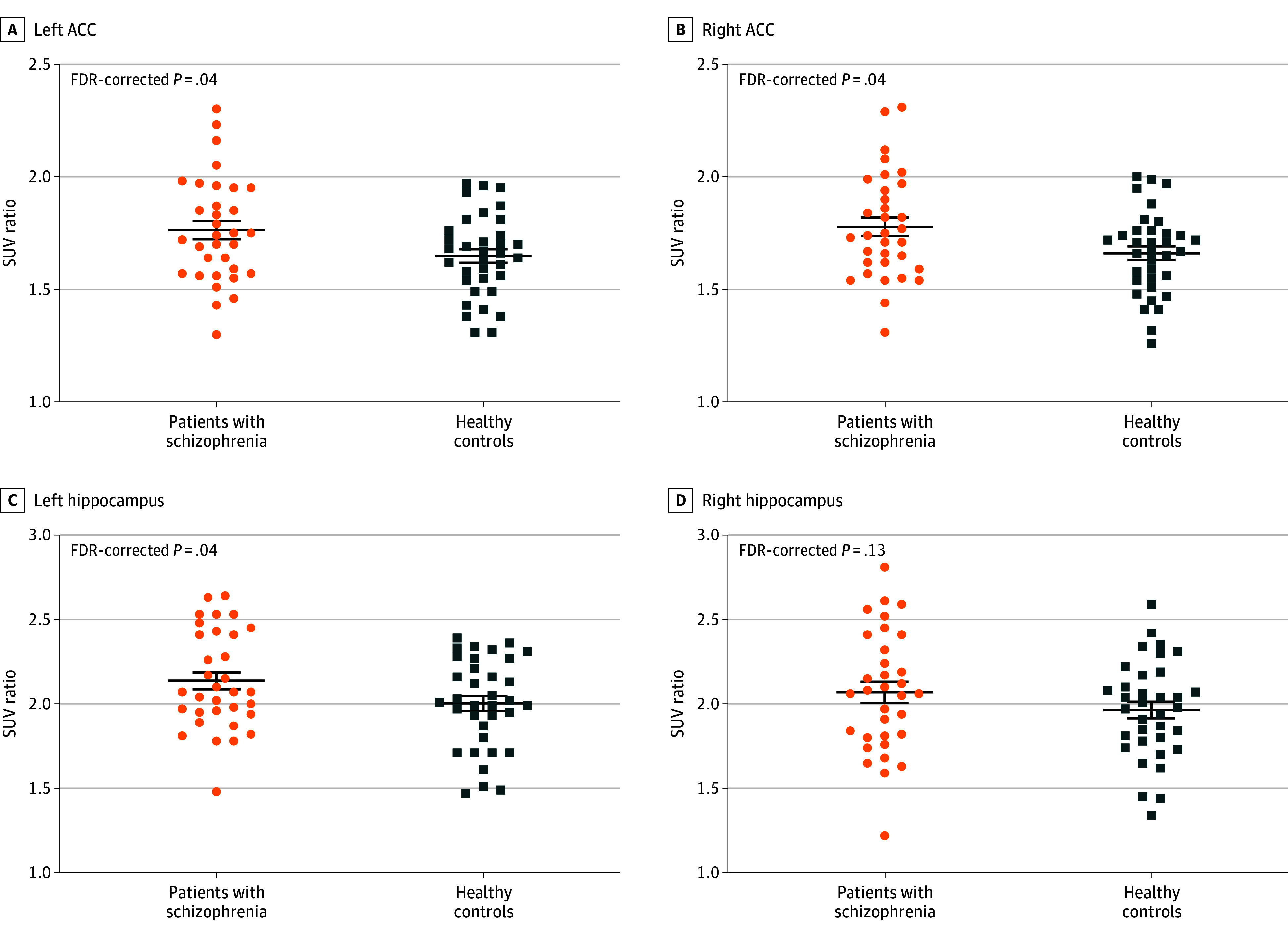
Group Comparison of Fluorine 18–Labeled THK5351 Retention in Bilateral Anterior Cingulate Cortex (ACC) and Hippocampus Standardized uptake value (SUV) ratios of the bilateral ACC and hippocampus were compared between patients with schizophrenia and healthy controls. Statistical significance was determined as a false discovery rate (FDR)–corrected *P* < .05. The center horizontal bars indicate means; the outer horizontal bars indicate 95% CIs.

**Figure 2.**
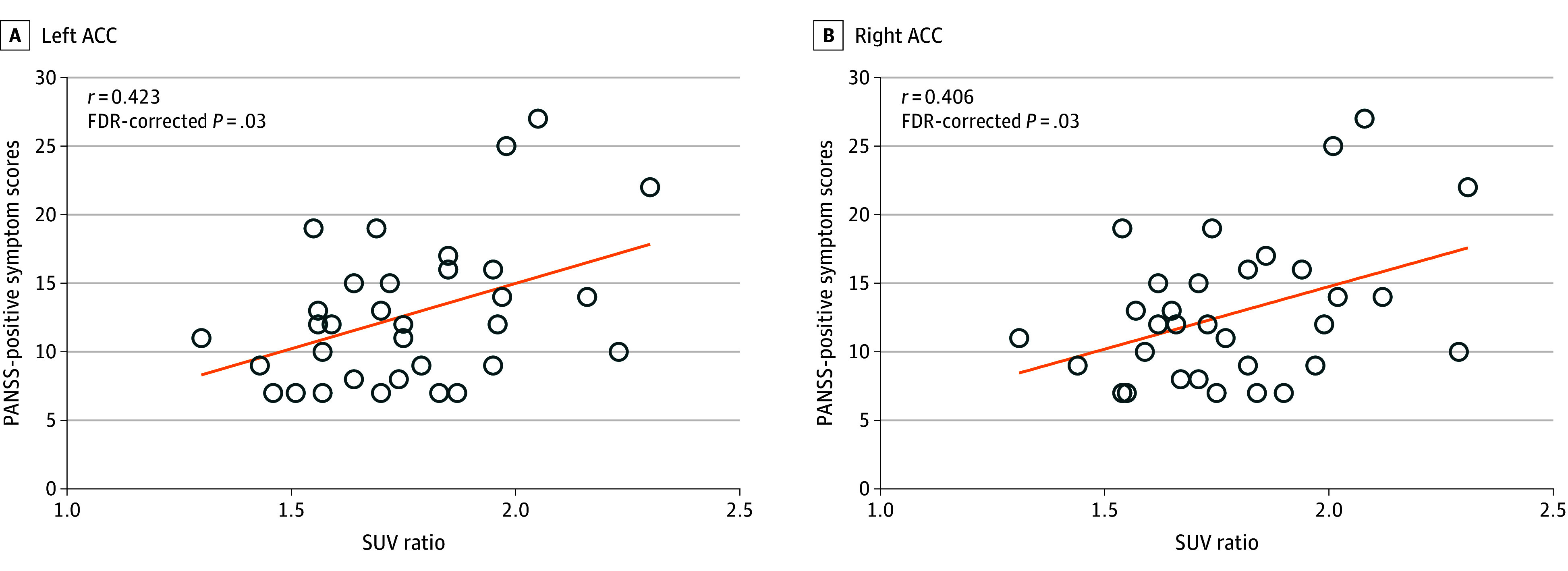
Correlation Between Fluorine 18–Labeled THK5351 Retention and Positive Symptom Severity in Patients With Schizophrenia Pearson correlation analysis between standardized uptake value (SUV) ratios of the bilateral anterior cingulate cortex (ACC) and positive symptom scores on the Positive and Negative Syndrome Scale (PANSS; range, 7-49, with higher scores indicating the greater severity of positive symptoms) was performed. Statistical significance was determined as a false discovery rate (FDR)–corrected *P* < .05. The orange lines indicate regression lines.

According to the group comparison of primary ROIs, patients with schizophrenia had significantly greater SUVrs in the bilateral ACC (left, *F* = 5.767 [FDR-corrected *P* = .04]; right, *F* = 5.977 [FDR-corrected *P* = .04]) and left hippocampus (*F* = 4.834 [FDR-corrected *P* = .04]) than healthy controls (Table 2 and Figure 1). There were positive correlations between the SUVrs in the bilateral ACC and the PANSS positive symptom scores (left, *r* = 0.423 [FDR-corrected *P* = .03]; right, *r* = 0.406 [FDR-corrected *P* = .03]) in patients with schizophrenia (Figure 2 and eTable 1 in Supplement 1). Repeated-measures ANOVA revealed trend-level group differences in the SUVrs in the secondary ROIs (eg, right parahippocampal gyrus, *F* = 3.387 [*P* = .07]) (eFigure 2 and eTable 2 in Supplement 1). There was no correlation between the duration of illness or olanzapine-equivalent dose of antipsychotics prescribed at the time of study participation and the SUVrs of the primary ROIs (eTable 3 in Supplement 1).

**Table 2.** Group Comparison Results of Fluorine 18–Labeled THK5351 Uptake in Primary ROIs

Primary ROI	SUV ratio, mean (SD)^a^		Statistical analysis^b^		
	Schizophrenia (n = 33)	Healthy controls (n = 35)	*F* statistic	*P* value	FDR-corrected *P* value
Left anterior cingulate cortex	176.3 (232.9)	164.8 (181.1)	5.767	.02^c^	.04^c^
Right anterior cingulate cortex	177.8 (233.3)	166.1 (182.7)	5.977	.02^c^	.04^c^
Left hippocampus	213.6 (289.8)	200.3 (265.6)	4.834	.03^c^	.04^c^
Right hippocampus	206.8 (355.4)	196.4 (286.9)	2.311	.13	.13

Abbreviations: FDR, false discovery rate; ROI, region of interest; SUV, standardized uptake value.

^a^ Data are multiplied by 10^−3^.

^b^ Analysis of covariance with age and sex as covariates.

^c^ Statistically significant at *P* < .05.

## Discussion

In this study, we investigated in vivo reactive astrocyte imaging using MAO-B–binding [^18^F]THK5351 PET to reveal the role of reactive astrocytes, which are involved in both glutamate imbalance and neuroinflammation, in schizophrenia pathophysiology. Patients with schizophrenia had elevated SUVrs in the bilateral ACC and left hippocampus compared with healthy controls. In addition, positive correlations between the SUVrs in the bilateral ACC and the PANSS-positive symptom scores were found in patients with schizophrenia. There were trend-level group differences in the SUVrs in other limbic regions investigated as secondary ROIs. The results of this study not only provide in vivo neuroimaging evidence of the role of reactive astrocytes in schizophrenia pathophysiology but also highlight the region-specific association between reactive astrocytes in the ACC and positive symptoms in patients with schizophrenia.

Our findings of elevated MAO-B–binding [^18^F]THK5351 uptake in patients with schizophrenia suggest a role for reactive astrocytes in the neurodevelopmental abnormalities of these patients. Astrocytes are known to be important in neurodevelopment and to play a critical role in synapse formation and function as well as neuronal survival and migration; thus, abnormal astrocytes can increase the vulnerability of the brain to neurodevelopmental disorders such as schizophrenia.^38,39^ Windrem et al^40^ showed that mice chimerized with induced pluripotent stem cells derived from patients with childhood-onset schizophrenia exhibited problems with glial cells, including astrocytes, suggesting that genetic abnormalities in schizophrenia produce abnormalities in astrocytes, which are critical for brain development and schizophrenia pathophysiology. Thus, the results of the present study support the vulnerability and neurodevelopmental model of schizophrenia and suggest the role of reactive astrocytes in schizophrenia pathophysiology by providing in vivo neuroimaging evidence of reactive astrocytes in patients with schizophrenia.

Furthermore, the results of this study provide integrative supporting evidence for the glutamate and immune hypotheses in schizophrenia pathophysiology. Previous studies aimed at measuring glutamate and/or glutamine levels in patients with schizophrenia^11,13^ have produced inconsistent results, which may be due to the lack of consideration of interacting systems other than glutamate. Microglial imaging studies using translocator protein PET to show neuroinflammation in patients with schizophrenia^16,18^ have also provided insufficient evidence that may be caused by subtle changes in microglial activity. Astrocytes not only play important roles in glutamate cycling and synaptic transmission but also undergo astrogliosis in reaction to neuroinflammation, as shown in neuroinflammatory diseases such as Wilson disease and multiple sclerosis.^2,6,25,41^ Therefore, the results of the present study support that both the glutamate and immune hypotheses can provide a more comprehensive understanding of the interaction between glutamate and the immune system in the pathophysiology of schizophrenia by revealing reactive astrocytes in vivo in patients with schizophrenia.

In the present study, elevated [^18^F]THK5351 uptake was detected in the bilateral ACC and left hippocampus of patients with schizophrenia compared with healthy controls. These findings are consistent with previous studies^23,42^ reporting that elevated astrocyte-, glutamate-, and immune-related genes and gene products were found in the postmortem brains of patients with schizophrenia. Previous neuroimaging studies targeting the glutamate system or neuroinflammation^13,14,16,28,43^ have also reported alterations in the ACC and hippocampus, which are known to be important brain regions involved in schizophrenia. In addition, an elevated SUVr of MAO-B–binding [^18^F]THK5351 in the bilateral ACC was positively correlated with positive symptom severity, as measured by the PANSS, in patients with schizophrenia. The ACC is one of the limbic cortices, and its role in fundamental cognitive processes, such as motivation, decision-making, and social cognition, which are impaired in patients with schizophrenia in relation to psychotic symptoms, has been highlighted.^44,45^ A previous study from Kim et al,^46^ which revealed that thalamocortical dysrhythmia represented by elevated resting-state theta phase–gamma amplitude coupling in the ACC of patients with schizophrenia spectrum disorder was positively correlated with symptom severity, also supported the association of reactive astrocytes in the ACC with positive symptoms.^2^

### Limitations

This study has several limitations. First, the current study participants were patients with schizophrenia who had more than 5 years of antipsychotic treatment, although there have been suggestions of an association between prolonged exposure to antipsychotics and increased MAO-B expression in animal studies.^47,48^ However, there have been no reports regarding the association between chronic antipsychotic exposure and MAO-B elevation in patients with schizophrenia, and a recent study^49^ suggested that MAO-B detected by PET imaging is a target for novel drug development in patients with schizophrenia.^23,50^ In addition, we did not find any correlation between the duration of illness or olanzapine-equivalent dose of antipsychotics prescribed at the time of study participation and the SUVrs of the primary ROIs. The current study results should be interpreted with caution because we did not perform this study in patients who were drug naive or with first-episode psychosis who may experience a minimal effect of antipsychotics on MAO-B expression. Second, because of the limitations of the MAO-B–detecting PET method, this study could only provide indirect evidence of reactive astrocytes in schizophrenia pathophysiology, as in previous animal, postmortem brain, and neuroimaging studies.^13,21,23,40^ Further studies with direct methods to investigate astrocytes in patients with schizophrenia are warranted to confirm the role of reactive astrocytes in the pathophysiology of schizophrenia. Third, the results of the present study are exploratory findings that need further validation in other studies due to the small effect size associated with the small sample size and other limitations mentioned above.

## Conclusions

This case-control study provides novel in vivo imaging evidence of reactive astrocyte involvement in the pathophysiology of schizophrenia. Considering the role of astrocytes in brain development, neurotransmission, and immune reactions, reactive astrocytes can be a strong biomarker for schizophrenia treatment. In particular, reactive astrocytes in the ACC may be a future target of neuromodulation therapeutics for the positive symptoms of schizophrenia. To support the findings of the present study, a more direct investigation of astrocytes generated by the reverse differentiation of induced pluripotent stem cells derived from patients with schizophrenia is needed.

## Notes

Supplement 1. **eMethods. **Sample Size Determination and Fluorine 18–Labeled THK 5351 Preparation **eTable 1.** Pearson Correlation Analysis Between the Altered Standardized Uptake Ratio of Fluorine 18– Labeled THK5351 in Primary Regions of Interest and Positive Symptom Severity in Patients With Schizophrenia **eTable 2.** Group Comparison Results of the Standardized Uptake Value Ratio of Fluorine 18– Labeled THK5351 in Secondary Regions of Interest **eTable 3.** Pearson Correlation Analysis Between Altered Standardized Uptake Ratio of Fluorine 18– Labeled THK5351 in Primary Regions of Interest and Olanzapine-Equivalent Dose of Antipsychotics as Well as Duration of Illness in Patients With Schizophrenia **eFigure 1.** Schematic Diagram of Fluorine 18– Labeled THK 5351 Positron Emission Tomography Analysis
**eFigure 2.** Group Comparison of Fluorine 18– Labeled THK5351 Standardized Uptake Value Ratios
eReferences

Supplement 2.
Data Sharing Statement
