# Supplementary material for: In Vivo Reactive Astrocyte Imaging in Patients With Schizophrenia Using Fluorine 18–Labeled THK5351
Source: JAMA Netw Open. 2024 May 9;7(5):e2410684. doi: 10.1001/jamanetworkopen.2024.10684 (PMC11082693; doi:10.1001/jamanetworkopen.2024.10684)
Supplement: Supplement 1. — eMethods. Sample Size Determination and Fluorine 18–Labeled THK 5351 Preparation eTable 1. Pearson Correlation Analysis Between the Altered Standardized Uptake Ratio of Fluorine 18– Labeled THK5351 in Primary Regions of Interest and Positive Symptom Severity in Patients With Schizophrenia eTable 2. Group Comparison Results of the Standardized Uptake Value Ratio of Fluorine 18– Labeled THK5351 in Secondary Regions of Interest eTable 3. Pearson Correlation Analysis Between Altered Standardized Uptake Ratio of Fluorine 18– Labeled THK5351 in Primary Regions of Interest and Olanzapine-Equivalent Dose of Antipsychotics as Well as Duration of Illness in Patients With Schizophrenia eFigure 1. Schematic Diagram of Fluorine 18– Labeled THK 5351 Positron Emission Tomography Analysis eFigure 2. Group Comparison of Fluorine 18– Labeled THK5351 Standardized Uptake Value Ratios eReferences [file jamanetwopen-e2410684-s001.pdf]

Supplementary Online Content

Kim M, Choi W, Choi S, et al. In vivo reactive astrocyte imaging in patients with schizophrenia using fluorine 18–labeled THK5351. *JAMA Netw Open*. 2024;7(5):e2410684. doi:10.1001/jamanetworkopen.2024.10684

eMethods. Sample Size Determination and Fluorine 18–Labeled THK 5351 Preparation

eTable 1. Pearson Correlation Analysis Between the Altered Standardized Uptake Ratio of Fluorine 18–Labeled THK5351 in Primary Regions of Interest and Positive Symptom Severity in Patients With Schizophrenia

eTable 2. Group Comparison Results of the Standardized Uptake Value Ratio of Fluorine 18–Labeled THK5351 in Secondary Regions of Interest

eTable 3. Pearson Correlation Analysis Between Altered Standardized Uptake Ratio of Fluorine 18–Labeled THK5351 in Primary Regions of Interest and Olanzapine-Equivalent Dose of Antipsychotics as Well as Duration of Illness in Patients With Schizophrenia

eFigure 1. Schematic Diagram of Fluorine 18–Labeled THK 5351 Positron Emission Tomography Analysis

eFigure 2. Group Comparison of Fluorine 18–Labeled THK5351 Standardized Uptake Value Ratios

eReferences

This supplementary material has been provided by the authors to give readers additional information about their work.

© 2024 Kim M et al. *JAMA Network Open*.

Methods. Sample Size Determination and Fluorine 18–Labeled THK 5351 Preparation

Sample size determination.

When performing an independent samples t test to yield an effect size d = 0.5 with two tailed, alpha error probability = 0.2, power (1-beta error probability) = 0.8, 37 samples are needed for each group. In addition, the sample size of a previous study using [^{18}F]THK5351 PET for investigating patients with cognitive disorders was approximately 35 for each group.[1] Thus, we investigated 33 patients with schizophrenia and 35 age- and sex-matched healthy control participants in this study.

[^{18}F]THK 5351 Preparation.

For radio thin-layer chromatography (TLC), instant thin-layer chromatography-silica gel (ITLC-SG) plates were obtained from the Pall Company (New York, USA). A Bioscan AR-2000 TLC Imaging Scanner (Washington, DC, USA) was used to perform the radio-TLC scan. A Gilson high-pressure liquid chromatography (HPLC) system (Middleton, USA) was used for purification and to check the purity of [^{18}F]THK5351. C18 and QMA Sep-Pak cartridges were obtained from Waters (Milford, USA). [^{18}F]Fluoride was produced by the 18O(p, n)18F reaction on 18O-enriched (97%) water using a 16.5 MeV proton beam generated by a GE PETTraceTM 800 cyclotron (Chicago, USA). The precursor of [^{18}F]THK5351 was purchased from FutureChem (Seoul, South Korea).

[^{18}F]THK5351 was prepared from its precursor (S)-2-(2-methylaminopyrid-5-yl)-6-[[2-(tetrahydro-2H-pyran-2-yloxy)-3-tosyloxy]propoxy]quinolone according to a previously reported method with slight modifications.[2] Briefly, an aqueous solution of [^{18}F]fluoride in 18O-enriched

© 2024 Kim M et al. *JAMA Network Open*.

water was captured on a QMA light SepPak cartridge and eluted with K2CO3/K222 solution (0.6 mL). Water was removed by azeotropic evaporation with acetonitrile at 90°C for 10 min. After being dried, [^{18}F]fluoride was reacted with the precursor (2.5 mg) in DMSO (1 mL) at 115°C for 10 min. After the reaction, 1 M HCl (0.6 mL) was added to the reaction mixture, and then the reaction mixture was neutralized with 1 M NaOAc solution (1.5 mL). The reaction mixture was purified by HPLC (column: Waters XBridge BEH C18 Prep Column, 5 µm, 10 x 250 mm; eluent: from 0% EtOH/water to 100% EtOH/water, for 30 min; 5 mL/min) to yield [^{18}F]THK5351 (radiochemical purity: 99.5%; radiochemical yield (EOS): 14.2±4.3%; molar activity: 201±34.4 GBq/µmol (n=3)).

© 2024 Kim M et al. *JAMA Network Open*.

eTable 1. Pearson Correlation Analysis Between the Altered Standardized Uptake Ratio of Fluorine 18–Labeled THK5351 in Primary Regions of Interest and Positive Symptom Severity in Patients With Schizophrenia

Clinical assessment	SUVr in primary ROIs	r	P	FDR-corrected P
PANSS positive symptoms	Left anterior cingulate cortex	0.423	0.01[a]	0.03[a]
	Right anterior cingulate cortex	0.406	0.02[a]	0.03[a]
	Left hippocampus	0.302	0.09	0.09

Abbreviations: PANSS, Positive and Negative Syndrome Scale, FDR, false discovery rate.
[a] Statistical significance at $p < 0.05$.

© 2024 Kim M et al. *JAMA Network Open*.

eTable 2. Group Comparison Results of the Standardized Uptake Value Ratio of Fluorine 18–Labeled THK5351 in Secondary Regions of Interest

Secondary ROIs	Schizophrenia (N = 33)	Healthy control (N = 35)	Statistical analysis[a]	
	SUVr[b]		F	P
Left posterior cingulate cortex	134.9 (155.6)	131.7 (134.0)	1.610	0.21
Right posterior cingulate cortex	131.4 (151.3)	128.5 (118.0)	1.482	0.23
Left parahippocampal gyrus	180.5 (218.3)	172.7 (205.3)	3.105	0.08
Right parahippocampal gyrus	176.8 (231.3)	168.3 (205.6)	3.387	0.07
Left amygdala	238.4 (330.2)	229.5 (285.9)	1.763	0.19
Right amygdala	244.4 (368.5)	230.6 (325.8)	3.214	0.08
Left insula	139.9 (197.1)	135.5 (157.4)	1.454	0.23
Right insula	138.0 (195.6)	134.5 (155.9)	0.922	0.34
Left nucleus accumbens	317.0 (457.7)	293.9 (575.1)	3.756	0.06
Right nucleus accumbens	318.5 (553.3)	293.1 (568.5)	3.707	0.06

[a] Analysis of covariance with age and sex as covariates.
[b] Data are multiplied by e-3.
Data are presented as the means (standard deviations).

© 2024 Kim M et al. *JAMA Network Open*.

eTable 3. Pearson Correlation Analysis Between Altered Standardized Uptake Ratio of Fluorine 18–Labeled THK5351 in Primary Regions of Interest and Olanzapine-Equivalent Dose of Antipsychotics as Well as Duration of Illness in Patients With Schizophrenia

Clinical assessment	SUVr in primary ROIs	r	P
Olanzapine equivalent dose of antipsychotics (mg/day)	Left anterior cingulate cortex	-0.181	0.31
	Right anterior cingulate cortex	-0.158	0.38
	Left hippocampus	-0.164	0.36
Duration of illness (months)	Left anterior cingulate cortex	0.126	0.49
	Right anterior cingulate cortex	0.113	0.53
	Left hippocampus	0.019	0.92

© 2024 Kim M et al. *JAMA Network Open*.

eFigure 1. Schematic Diagram of Fluorine 18–Labeled THK 5351 Positron Emission Tomography Analysis

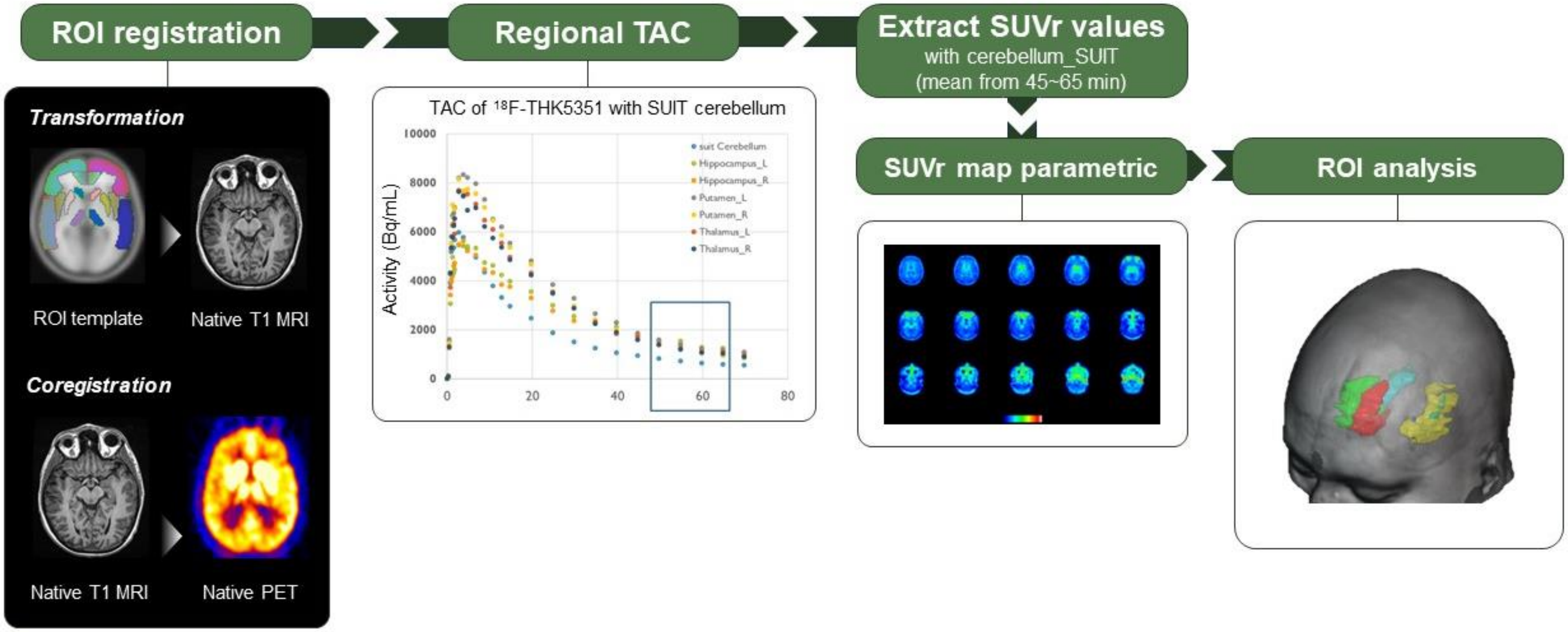

The predefined regions of interest (ROIs) were transformed into PET standard space using the deformation matrix calculated from PET-coregistered T1 magnetic resonance images, and standardized uptake values were extracted for all PET frames to check the time activity curve (TAC) in each ROI. Standardized uptake value ratios (SUVrs) were calculated with a sum of 45 to 65 min postinjection frames using the mean radioactivity of the inferior cerebellar ROI obtained by the SUIT procedure as a reference.

© 2024 Kim M et al. *JAMA Network Open*.

eFigure 2. Group Comparison of Fluorine 18–Labeled THK5351 Standardized Uptake Value Ratios

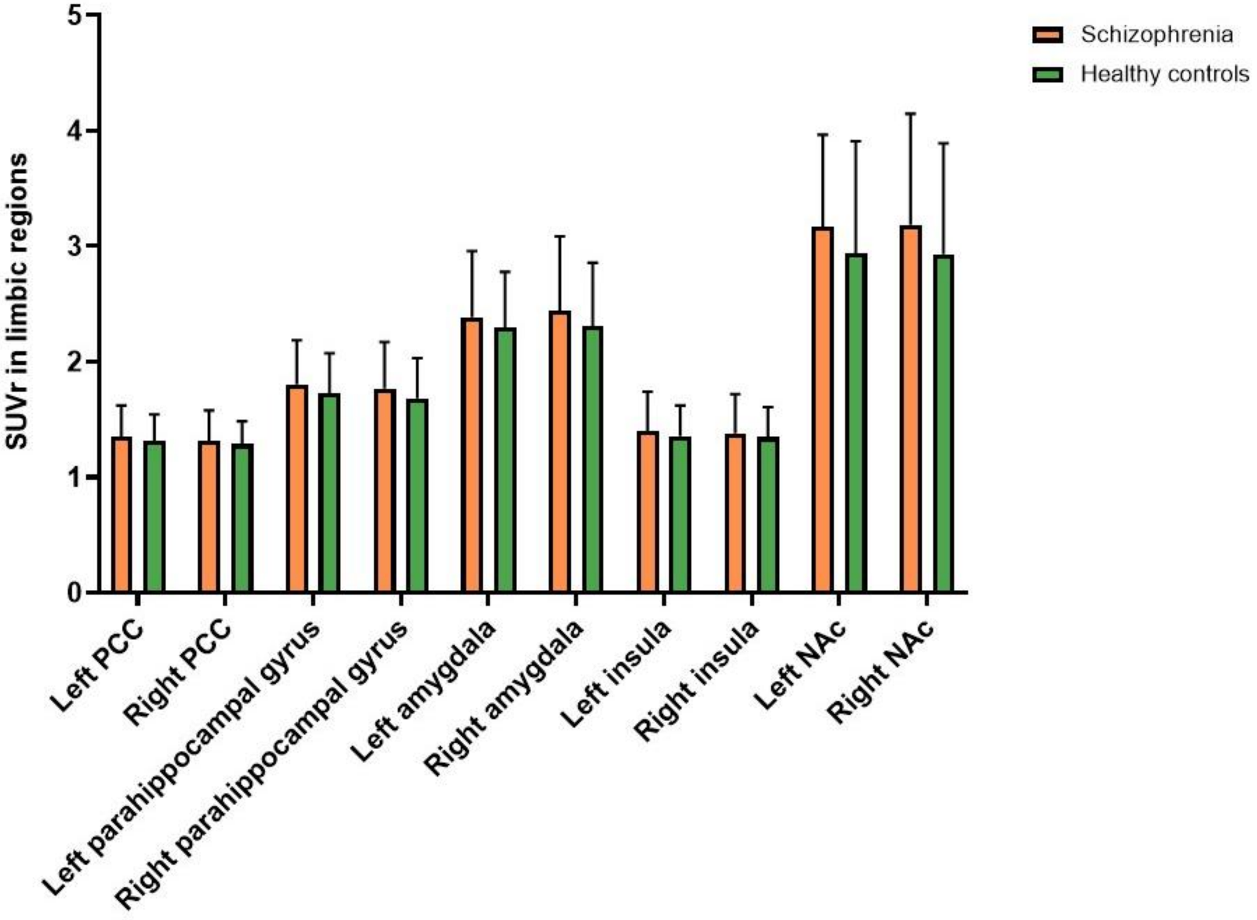

Abbreviations: PCC, posterior cingulate cortex; NAc, nucleus accumbens.

© 2024 Kim M et al. *JAMA Network Open*.

eReferences

1. Jeong HJ, Lee H, Lee SY, et al. [^{18}F]THK5351 PET Imaging in Patients with Mild Cognitive Impairment. *J Clin Neurol.* 2020;16(2):202-214.

2. Okamura N, Furumoto S, Harada R, et al. Novel ^{18}F-labeled arylquinoline derivatives for noninvasive imaging of tau pathology in Alzheimer disease. *J Nucl Med.* 2013;54(8):1420-1427.

© 2024 Kim M et al. *JAMA Network Open*.
