# Supplementary material for: In Vivo Reactive Astrocyte Imaging in Patients With Schizophrenia Using Fluorine 18–Labeled THK5351
Source: JAMA Netw Open. 2024 May 9;7(5):e2410684. doi: 10.1001/jamanetworkopen.2024.10684 (PMC11082693; doi:10.1001/jamanetworkopen.2024.10684)
Supplement: Supplement 2. — Data Sharing Statement [file jamanetwopen-e2410684-s002.pdf]

Data Sharing Statement

Kim. In Vivo Reactive Astrocyte Imaging in Patients With Schizophrenia Using Fluorine 18–Labeled THK5351. *JAMA Netw Open*. Published May 09, 2024.
doi:10.1001/jamanetworkopen.2024.10684

Data
Data available: No

Additional Information
Explanation for why data not available: The data that support the results of this study are available from the corresponding author upon reasonable request. The data are not publicly available because they contain information that might compromise the privacy of the research participants.
